# *N*′-(4-Ethyl­cyclo­hexyl­idene)-5-fluoro-3-phenyl-1*H*-indole-2-carbohydrazide

**DOI:** 10.1107/S1600536813020394

**Published:** 2013-07-27

**Authors:** Mehmet Akkurt, Sevim Türktekin Çelikesir, Gökçe Cihan Üstündağ, Gültaze Çapan, Orhan Büyükgüngör

**Affiliations:** a Department of Physics, Faculty of Sciences, Erciyes University, 38039 Kayseri, Turkey; b Department of Pharmaceutical Chemistry, Faculty of Pharmacy, Istanbul University, 34116 Beyazit, Istanbul, Turkey; c Department of Physics, Faculty of Arts and Sciences, Ondokuz Mayıs University, 55139 Samsun, Turkey

## Abstract

The title compound, C_23_H_24_FN_3_O, crystallizes with two independent mol­ecules (I and II) in the asymmetric unit. These pairs of mol­ecules are linked to each other as N—H⋯O dimers with an *R*_2_^2^(10) motif. Furthermore, the crystal structure also exhibits C—H⋯π inter­actions. The atoms of the ethyl group in mol­ecule I are disordered over two sites with an occupancy ratio of 0.817 (6):0.183 (6).

## Related literature

For the anti­tubercular and anti­viral activity of variously substituted *N*-(1-thia-4-aza­spiro­[4.5]dec-4-yl)carboxamides, see: Cihan-Üstündağ & Çapan (2012 ▶); Göktas *et al.* (2012 ▶). For similar structures, see: Çelikesir *et al.* (2013*a* ▶,*b* ▶). For puckering analysis, see: Cremer & Pople (1975 ▶). For the graph-set analysis of hydrogen bonding, see: Bernstein *et al.* (1995 ▶).
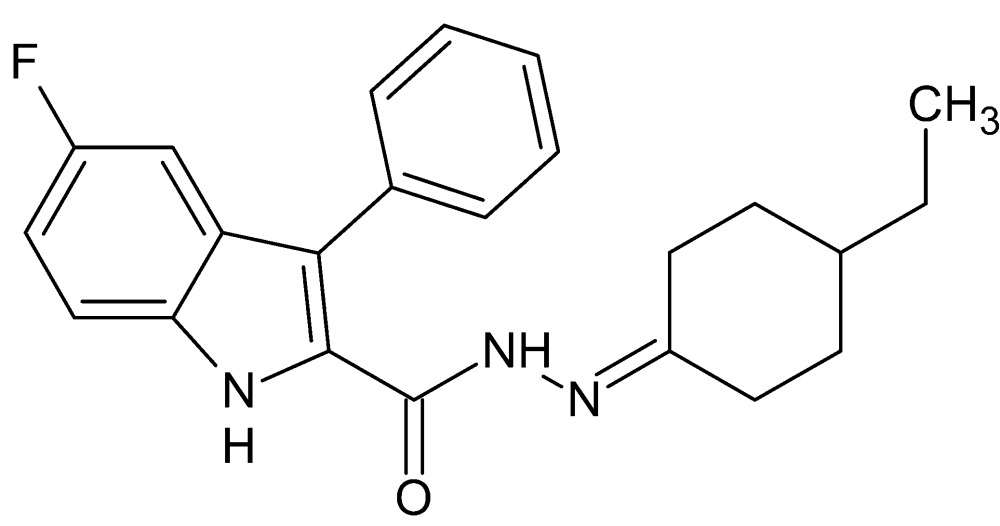


## Experimental

### 

#### Crystal data

C_23_H_24_FN_3_O
*M* *_r_* = 377.45
Triclinic, 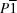

*a* = 11.8121 (5) Å
*b* = 13.3802 (5) Å
*c* = 15.5693 (6) Å
α = 114.328 (3)°
β = 95.642 (3)°
γ = 110.434 (3)°
*V* = 2014.85 (17) Å^3^
*Z* = 4
Mo *K*α radiation
μ = 0.08 mm^−1^
*T* = 296 K
0.55 × 0.48 × 0.37 mm

#### Data collection

Stoe IPDS 2 diffractometer
Absorption correction: integration (*X-RED32*; Stoe & Cie, 2002 ▶) *T*_min_ = 0.955, *T*_max_ = 0.969
30678 measured reflections
8272 independent reflections
5564 reflections with *I* > 2σ(*I*)
*R*_int_ = 0.108

#### Refinement

*R*[*F* ^2^ > 2σ(*F* ^2^)] = 0.067
*wR*(*F* ^2^) = 0.180
*S* = 1.02
8272 reflections
528 parameters
8 restraints
H atoms treated by a mixture of independent and constrained refinement
Δρ_max_ = 0.35 e Å^−3^
Δρ_min_ = −0.19 e Å^−3^

### 

Data collection: *X-AREA* (Stoe & Cie, 2002 ▶); cell refinement: *X-AREA*; data reduction: *X-RED32* (Stoe & Cie, 2002 ▶); program(s) used to solve structure: *SHELXS97* (Sheldrick, 2008 ▶); program(s) used to refine structure: *SHELXL97* (Sheldrick, 2008 ▶); molecular graphics: *ORTEP-3 for Windows* (Farrugia, 2012 ▶); software used to prepare material for publication: *WinGX* (Farrugia, 2012 ▶) and *PLATON* (Spek, 2009 ▶).

## Supplementary Material

Crystal structure: contains datablock(s) global, I. DOI: 10.1107/S1600536813020394/sj5346sup1.cif

Structure factors: contains datablock(s) I. DOI: 10.1107/S1600536813020394/sj5346Isup2.hkl

Supplementary material file. DOI: 10.1107/S1600536813020394/sj5346Isup3.cml

Additional supplementary materials:  crystallographic information; 3D view; checkCIF report

## Figures and Tables

**Table 1:** Hydrogen-bond geometry (Å, °) *Cg*1, *Cg*3, *Cg*5 and *Cg*7 are the centroids of the N1/C1/C6/C7/C14, C8–C13, N4/C24/C29/C30/C37 and C31–C36 rings, respectively.

*D*—H⋯*A*	*D*—H	H⋯*A*	*D*⋯*A*	*D*—H⋯*A*
N1—H1*N*⋯O2	0.85 (3)	2.00 (3)	2.836 (3)	168 (3)
N4—H4*N*⋯O1	0.85 (3)	2.08 (3)	2.887 (3)	160 (2)
C9—H9⋯*Cg*1^i^	0.93	2.81	3.636 (3)	149
C17—H17*A*⋯*Cg*3	0.97	2.87	3.820 (3)	165
C40—H40*A*⋯*Cg*7	0.97	2.79	3.727 (4)	163
C40—H40*B*⋯*Cg*5^ii^	0.97	2.65	3.595 (4)	163

Symmetry codes: (i) 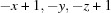; (ii) 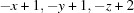.

**Fig. 1.**
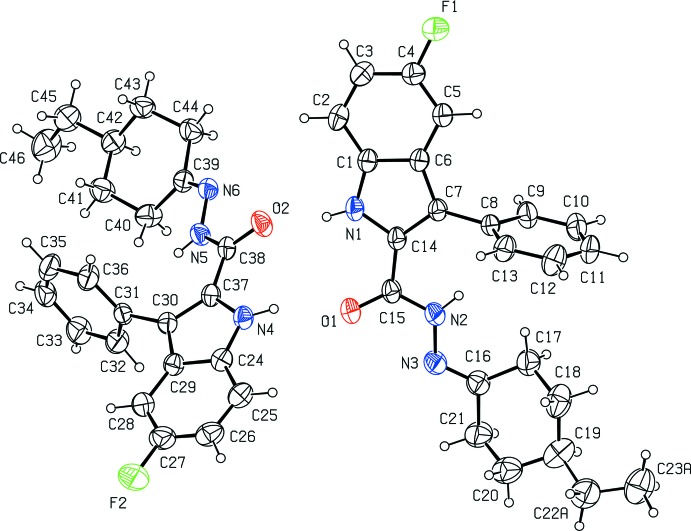
View of the pairs of molecules I and II of the title compound in the asymmetric unit. Displacement ellipsoids for non-H atoms are drawn at the 30% probability level.

**Fig. 2.**
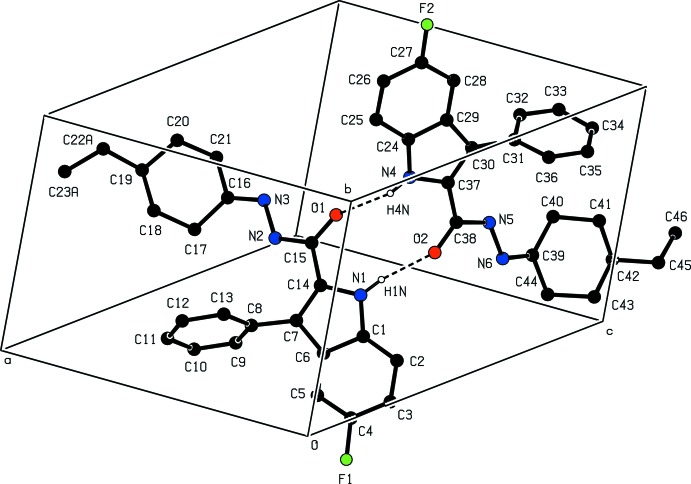
View of the N—H···O dimer between the pairs of molecules I and II in the asymmetric unit. H atoms not participating in hydrogen bonding have been omitted for clarity.

## supplementary crystallographic information

### Comment

On reaction with mercaptoalkanoic acids, cyclohexylidenehydrazides smoothly afford *N*-(1-thia-3-oxo-4-azaspiro[4.5]dec-4-yl)carboxamides with promising antituberculosis (Cihan-Üstündağ & Çapan, 2012) and antiviral (Göktas *et al.*, 2012) activity. We have recently reported on the crystal structures of two such precursors (Türktekin-Çelikesir *et al.*, 2013*a*,*b*). We herein report the X-ray diffraction analysis of the title compound.

In the title compound, (Fig. 1), the asymmetric unit contains two crystallographically independently molecules whose cyclohexane rings adopt chair conformations [the puckering parameters (Cremer & Pople, 1975) are Q_T_ = 0.507 (4) Å, θ = 4.0 (6)°, φ = 277 (7)° for molecule I (with N1), and Q_T_ = 0.531 (4) Å, θ = 12.5 (4) °, φ = 205.2 (18) ° for molecule II (with N4)].

The indole ring systems of both molecules I and II are essentially planar [maximum deviations are 0.026 (4) Å for C3 in molecule I and 0.028 (2) Å for C30 in molecule II]. The indole ring systems of molecules I and II make dihedral angles of 77.93 (12) and 77.66 (14)° with their phenyl rings, respectively.

In the crystal, pairs of molecules I and II in the asymmetric unit are linked to each other, forming N—H···O dimers (Table 1, Fig. 2), with the *R*_2_^2^(10) ring motif (Bernstein *et al.*, 1995). In addition, C—H···π interactions (Table 1) contribute to the stabilization of the crystal structure.

### Experimental

A mixture of 5-fluoro-3-phenyl-1*H*-indole-2-carbohydrazide (0.005 mol) and 4-ethyl cyclohexanone (0.007 mol) was refluxed in 15 ml absolute ethanol for 5 h. The precipitate obtained on cooling was purified by recrystallization from an ethanol-water mixture to afford colorless prisms.

[Yield: 79%, m.p.: 451.0- 452.5 K]. IR(KBr): υ_max_ 3346, 3235 (N—H), 1654 (C=O) cm^-1. 1^H-NMR (DMSO-d_6_/500 MHz): δ 0.84 (t, 3H, J= 7.2 Hz, 4-CH_2_CH_3_-cyc.*), 1.13 (br. d, 1H,J=6.8 Hz, CH/CH_2_-cyc.), 1.19 (br. t, 2H, J=6.6 Hz, 4-CH_2_CH_3_-cyc.), 1.33 (s, 1H, CH/ CH_2_-cyc.), 1.48 (br. s, 1H, CH/CH_2_-cyc.), 1.61 (d, 2H, J=12.7 Hz, CH/CH_2_-cyc.), 1.70–1.90 (m, 2H, CH/CH_2_-cyc.), 2.13 (s, 1H, CH/CH_2_-cyc.), 2.30 (s, 1H, CH/CH_2_-cyc.), 7.12 (br. t, 2H, J= 8.8 Hz, H4, H6-ind.), 7.42–7.51 (m, 6H, H7, 3-C_6_H_5_-ind.), 9.44 (s, 1H, CONH), 12.03 (s, 1H, NH) p.p.m.. ^13^C-NMR(APT, DMSO-d_6_/125 MHz): δ 12.12 (4-CH_2_CH_3_-cyc.), 26.06 (CH_2_-cyc.), 28.79 (4-CH_2_CH_3_-cyc.), 31.88 (CH_2_-cyc.), 32.86 (CH_2_-cyc.), 34.65 (CH_2_-cyc.), 38.32 (CH-cyc.), 104.64 (d, J=23.5 Hz, C4-ind.), 113.50 (d, C6-ind.), 114.40 (C7-ind.), 118.00 (C3-ind.), 126.80 (C3a-ind.), 128.54 (3-C_6_H_5_ (C4)-ind.), 129.68 (3-C_6_H_5_(C3,C5)-ind.), 129.91 (C2-ind.), 130.90 (3-C_6_H_5_(C2,C6)-ind.), 133.03** (C7a-ind.), 134.02** (3-C_6_H_5_(C1)-ind.), 158.24 (C=N), 158.30 (d, J=233.6 Hz, C5-ind.), 162.11 (C=O) p.p.m.. MS (APCI+) m/*z* (%) 378 ((*M*+H)^+^, 100), (APCI–) m/*z* (%) 376 ((M—H)^-^, 100). Analysis calculated for C_23_H_24_FN_3_O: C 73.19, H 6.41, N 11.13%. Found: C 72.84, H 6.68, N 10.93%.(*cyc.=cyclohexylidene, br.=broad, ind.=indole, **interchangeable).

### Refinement

H atoms bonded to C atoms were positioned geometrically with C—H = 0.93 - 0.98 Å, and refined using a riding model with *U*_iso_(H) = 1.2 or 1.5*U*_eq_(C). The H atoms of the four amide groups were found in a difference Fourier map, and refined freely. The atoms of the ethyl group in molecule I (with N1) are disordered over two sites (with the suffixes A and B) with the refined occupancy ratio of 0.817 (6):0.183 (6). The atoms of the disordered ethyl group in molecule I were set to equal each other by an EADP instruction. Four poorly fitted reflections (1 - 3 1), (2 - 1 2), (1 - 3 3) and (3 - 2 1) were omitted from the refinemet.

### Crystal data

**Table:**

C_23_H_24_FN_3_O	*Z* = 4
*M**_r_* = 377.45	*F*(000) = 800
Triclinic, *P*1	*D*_x_ = 1.244 Mg m^−^^3^
Hall symbol: -P 1	Mo *K*α radiation, λ = 0.71073 Å
*a* = 11.8121 (5) Å	Cell parameters from 34554 reflections
*b* = 13.3802 (5) Å	θ = 2.0–27.3°
*c* = 15.5693 (6) Å	µ = 0.08 mm^−^^1^
α = 114.328 (3)°	*T* = 296 K
β = 95.642 (3)°	Prism, colourless
γ = 110.434 (3)°	0.55 × 0.48 × 0.37 mm
*V* = 2014.85 (17) Å^3^	

### Data collection

**Table:**

Stoe IPDS 2 diffractometer	8272 independent reflections
Radiation source: sealed X-ray tube, 12 x 0.4 mm long-fine focus	5564 reflections with *I* > 2σ(*I*)
Plane graphite monochromator	*R*_int_ = 0.108
Detector resolution: 6.67 pixels mm^-1^	θ_max_ = 26.5°, θ_min_ = 2.3°
ω–scans	*h* = −14→14
Absorption correction: integration (*X-RED32*; Stoe & Cie, 2002)	*k* = −16→16
*T*_min_ = 0.955, *T*_max_ = 0.969	*l* = −19→19
30678 measured reflections	

### Refinement

**Table:**

Refinement on *F*^2^	8 restraints
Least-squares matrix: full	H atoms treated by a mixture of independent and constrained refinement
*R*[*F*^2^ > 2σ(*F*^2^)] = 0.067	W = 1/[Σ^2^(*F*O^2^) + (0.083*P*)^2^ + 0.3779*P*] WHERE *P* = (*F*O^2^ + 2*F*C^2^)/3
*wR*(*F*^2^) = 0.180	(Δ/σ)_max_ = 0.001
*S* = 1.02	Δρ_max_ = 0.35 e Å^−^^3^
8272 reflections	Δρ_min_ = −0.19 e Å^−^^3^
528 parameters	

### Special details

**Table:**

Geometry. Bond distances, angles *etc*. have been calculated using the rounded fractional coordinates. All su's are estimated from the variances of the (full) variance-covariance matrix. The cell e.s.d.'s are taken into account in the estimation of distances, angles and torsion angles
Refinement. Refinement on *F*^2^ for ALL reflections except those flagged by the user for potential systematic errors. Weighted *R*-factors *wR* and all goodnesses of fit *S* are based on *F*^2^, conventional *R*-factors *R* are based on *F*, with *F* set to zero for negative *F*^2^. The observed criterion of *F*^2^ > σ(*F*^2^) is used only for calculating -*R*-factor-obs *etc*. and is not relevant to the choice of reflections for refinement. *R*-factors based on *F*^2^ are statistically about twice as large as those based on *F*, and *R*-factors based on ALL data will be even larger.

### Fractional atomic coordinates and isotropic or equivalent isotropic displacement parameters (Å^2^)

**Table:**

	*x*	*y*	*z*	*U*_iso_*/*U*_eq_	Occ. (<1)
F1	0.05143 (16)	−0.22445 (17)	0.21734 (13)	0.0940 (6)	
O1	0.59795 (19)	0.40150 (17)	0.65907 (12)	0.0752 (7)	
N1	0.3715 (2)	0.2028 (2)	0.53565 (15)	0.0612 (7)	
N2	0.7073 (2)	0.3010 (2)	0.58120 (15)	0.0662 (8)	
N3	0.8220 (2)	0.3863 (2)	0.65017 (14)	0.0699 (8)	
C1	0.2761 (2)	0.0988 (2)	0.46077 (16)	0.0578 (8)	
C2	0.1458 (3)	0.0503 (3)	0.4466 (2)	0.0717 (10)	
C3	0.0724 (3)	−0.0574 (3)	0.3636 (2)	0.0743 (10)	
C4	0.1284 (3)	−0.1150 (3)	0.29687 (19)	0.0686 (9)	
C5	0.2541 (2)	−0.0691 (2)	0.30711 (17)	0.0623 (8)	
C6	0.3310 (2)	0.0409 (2)	0.39164 (16)	0.0542 (8)	
C7	0.4643 (2)	0.1138 (2)	0.42771 (15)	0.0524 (7)	
C8	0.5555 (2)	0.0856 (2)	0.37421 (15)	0.0521 (7)	
C9	0.5933 (3)	−0.0018 (2)	0.3731 (2)	0.0710 (10)	
C10	0.6731 (3)	−0.0321 (3)	0.3197 (2)	0.0847 (11)	
C11	0.7165 (3)	0.0241 (3)	0.2664 (2)	0.0914 (12)	
C12	0.6810 (3)	0.1118 (4)	0.2665 (2)	0.0949 (14)	
C13	0.6016 (3)	0.1429 (3)	0.3202 (2)	0.0717 (10)	
C14	0.4853 (2)	0.2117 (2)	0.51692 (16)	0.0547 (8)	
C15	0.6011 (2)	0.3143 (2)	0.59215 (16)	0.0577 (8)	
C16	0.9194 (3)	0.3713 (3)	0.63047 (18)	0.0691 (9)	
C17	0.9279 (3)	0.2770 (3)	0.5408 (2)	0.0833 (11)	
C18	1.0327 (4)	0.3371 (4)	0.5030 (3)	0.1061 (18)	
C19	1.1560 (3)	0.4280 (4)	0.5781 (3)	0.1004 (16)	
C20	1.1385 (3)	0.5181 (4)	0.6656 (3)	0.1005 (16)	
C21	1.0427 (3)	0.4600 (3)	0.7075 (2)	0.0902 (11)	
C22A	1.2593 (5)	0.4974 (5)	0.5436 (4)	0.110 (2)	0.817 (6)
C23A	1.3041 (6)	0.4198 (6)	0.4711 (5)	0.126 (3)	0.817 (6)
C23B	1.366 (2)	0.508 (2)	0.537 (2)	0.126 (3)	0.183 (6)
C22B	1.2352 (13)	0.4138 (18)	0.5038 (15)	0.110 (2)	0.183 (6)
F2	0.6710 (2)	1.04361 (17)	0.94884 (19)	0.1217 (9)	
O2	0.31889 (19)	0.31546 (16)	0.71583 (12)	0.0730 (6)	
N4	0.4818 (2)	0.5522 (2)	0.76600 (14)	0.0581 (7)	
N5	0.2973 (2)	0.37572 (19)	0.86894 (15)	0.0569 (7)	
N6	0.21921 (19)	0.26057 (17)	0.85033 (14)	0.0580 (7)	
C24	0.5392 (2)	0.6749 (2)	0.80253 (17)	0.0586 (8)	
C25	0.6219 (3)	0.7449 (3)	0.7690 (2)	0.0719 (10)	
C26	0.6642 (3)	0.8682 (3)	0.8203 (2)	0.0820 (11)	
C27	0.6270 (3)	0.9203 (3)	0.9024 (3)	0.0831 (11)	
C28	0.5475 (3)	0.8556 (3)	0.9381 (2)	0.0725 (10)	
C29	0.5031 (2)	0.7290 (2)	0.88695 (17)	0.0569 (8)	
C30	0.4220 (2)	0.6328 (2)	0.90166 (15)	0.0529 (7)	
C31	0.3655 (2)	0.6474 (2)	0.98483 (15)	0.0526 (7)	
C32	0.4393 (3)	0.6817 (3)	1.07581 (18)	0.0729 (10)	
C33	0.3872 (3)	0.6855 (3)	1.15265 (19)	0.0841 (10)	
C34	0.2624 (3)	0.6584 (3)	1.1401 (2)	0.0803 (10)	
C35	0.1891 (3)	0.6286 (3)	1.0519 (2)	0.0867 (11)	
C36	0.2405 (3)	0.6232 (3)	0.97499 (19)	0.0749 (10)	
C37	0.4113 (2)	0.5258 (2)	0.82567 (15)	0.0521 (7)	
C38	0.3391 (2)	0.3971 (2)	0.79784 (15)	0.0528 (8)	
C39	0.1917 (2)	0.2488 (2)	0.92415 (17)	0.0579 (8)	
C40	0.2397 (3)	0.3436 (3)	1.03009 (18)	0.0736 (10)	
C41	0.1395 (3)	0.3362 (3)	1.0833 (2)	0.0788 (10)	
C42	0.0615 (3)	0.2083 (3)	1.0657 (2)	0.0745 (10)	
C43	0.0033 (3)	0.1244 (3)	0.9559 (2)	0.0751 (10)	
C44	0.1045 (3)	0.1235 (2)	0.9033 (2)	0.0770 (10)	
C45	−0.0340 (3)	0.2019 (3)	1.1234 (2)	0.0901 (12)	
C46	0.0227 (4)	0.2544 (4)	1.2315 (3)	0.1145 (16)	
H2	0.11020	0.08990	0.49220	0.0860*	
H1N	0.362 (2)	0.246 (2)	0.5898 (19)	0.062 (7)*	
H5	0.28770	−0.10920	0.26000	0.0750*	
H9	0.56410	−0.04090	0.40930	0.0850*	
H10	0.69740	−0.09100	0.32000	0.1020*	
H11	0.77020	0.00340	0.22990	0.1090*	
H12	0.71090	0.15030	0.23000	0.1130*	
H2N	0.693 (2)	0.237 (3)	0.534 (2)	0.072 (8)*	
H3	−0.01470	−0.09200	0.35160	0.0890*	
H17B	0.94590	0.21980	0.55600	0.1000*	
H18A	1.04670	0.27370	0.45240	0.1270*	
H18B	1.00290	0.37680	0.47230	0.1270*	
H19	1.18950	0.38310	0.60100	0.1200*	
H20A	1.11240	0.56930	0.64710	0.1200*	
H20B	1.21860	0.56970	0.71610	0.1200*	
H21A	1.07410	0.41830	0.73490	0.1080*	
H21B	1.03000	0.52250	0.76020	0.1080*	
H22A	1.22650	0.53750	0.51510	0.1310*	0.817 (6)
H22B	1.33040	0.55990	0.60040	0.1310*	0.817 (6)
H23A	1.36860	0.46880	0.45370	0.1900*	0.817 (6)
H23B	1.23510	0.35960	0.41340	0.1900*	0.817 (6)
H23C	1.33770	0.38050	0.49880	0.1900*	0.817 (6)
H13	0.57860	0.20270	0.32020	0.0860*	
H17A	0.84800	0.23270	0.49020	0.1000*	
H22C	1.23840	0.33610	0.48320	0.1310*	0.183 (6)
H22D	1.19020	0.41020	0.44620	0.1310*	0.183 (6)
H23D	1.39350	0.50710	0.48030	0.1900*	0.183 (6)
H23E	1.42060	0.49200	0.57380	0.1900*	0.183 (6)
H23F	1.36940	0.58660	0.57710	0.1900*	0.183 (6)
H4N	0.498 (2)	0.498 (2)	0.7245 (19)	0.065 (7)*	
H5N	0.314 (2)	0.436 (2)	0.9156 (14)	0.051 (7)*	
H25	0.64700	0.70880	0.71390	0.0860*	
H26	0.71830	0.91740	0.79960	0.0990*	
H28	0.52420	0.89380	0.99360	0.0870*	
H32	0.52510	0.70250	1.08560	0.0870*	
H33	0.43770	0.70670	1.21280	0.1010*	
H34	0.22720	0.66000	1.19130	0.0960*	
H35	0.10430	0.61190	1.04360	0.1040*	
H36	0.18950	0.60290	0.91540	0.0900*	
H40A	0.27480	0.42330	1.03430	0.0880*	
H40B	0.30720	0.33500	1.06280	0.0880*	
H41A	0.08380	0.36550	1.06210	0.0940*	
H41B	0.17940	0.38920	1.15330	0.0940*	
H42	0.12010	0.18130	1.08810	0.0890*	
H43A	−0.04730	0.04260	0.94380	0.0900*	
H43B	−0.05160	0.15130	0.93010	0.0900*	
H44A	0.15310	0.08770	0.92370	0.0920*	
H44B	0.06490	0.07330	0.83300	0.0920*	
H45A	−0.08110	0.24500	1.11400	0.1080*	
H45B	−0.09300	0.11760	1.09730	0.1080*	
H46A	0.08040	0.33820	1.25830	0.1370*	
H46B	0.06690	0.21030	1.24170	0.1370*	
H46C	−0.04290	0.24820	1.26350	0.1370*	

### Atomic displacement parameters (Å^2^)

**Table:**

	*U*^11^	*U*^22^	*U*^33^	*U*^12^	*U*^13^	*U*^23^
F1	0.0663 (10)	0.0881 (12)	0.0889 (11)	0.0211 (9)	0.0103 (8)	0.0211 (9)
O1	0.0809 (13)	0.0725 (11)	0.0617 (10)	0.0379 (10)	0.0299 (9)	0.0169 (9)
N1	0.0721 (14)	0.0673 (13)	0.0537 (11)	0.0388 (12)	0.0331 (10)	0.0267 (10)
N2	0.0667 (14)	0.0652 (14)	0.0505 (11)	0.0296 (12)	0.0162 (10)	0.0125 (10)
N3	0.0718 (15)	0.0743 (14)	0.0499 (10)	0.0305 (12)	0.0137 (10)	0.0194 (10)
C1	0.0628 (15)	0.0659 (15)	0.0558 (12)	0.0330 (13)	0.0259 (11)	0.0323 (11)
C2	0.0692 (18)	0.092 (2)	0.0718 (16)	0.0448 (16)	0.0382 (14)	0.0425 (15)
C3	0.0604 (17)	0.089 (2)	0.0780 (17)	0.0322 (16)	0.0249 (14)	0.0426 (16)
C4	0.0621 (17)	0.0685 (16)	0.0665 (15)	0.0248 (14)	0.0164 (12)	0.0279 (13)
C5	0.0657 (16)	0.0654 (15)	0.0575 (13)	0.0318 (13)	0.0246 (11)	0.0264 (12)
C6	0.0584 (14)	0.0615 (14)	0.0534 (12)	0.0316 (12)	0.0239 (10)	0.0302 (11)
C7	0.0598 (14)	0.0562 (13)	0.0493 (11)	0.0292 (12)	0.0238 (10)	0.0271 (10)
C8	0.0539 (13)	0.0541 (13)	0.0461 (10)	0.0241 (11)	0.0194 (9)	0.0203 (9)
C9	0.089 (2)	0.0674 (16)	0.0799 (16)	0.0468 (16)	0.0427 (15)	0.0405 (14)
C10	0.091 (2)	0.0739 (19)	0.100 (2)	0.0515 (18)	0.0443 (18)	0.0340 (17)
C11	0.084 (2)	0.101 (2)	0.087 (2)	0.050 (2)	0.0496 (17)	0.0290 (18)
C12	0.099 (2)	0.128 (3)	0.093 (2)	0.059 (2)	0.0607 (19)	0.068 (2)
C13	0.0769 (18)	0.0859 (19)	0.0782 (16)	0.0440 (16)	0.0393 (14)	0.0506 (15)
C14	0.0636 (15)	0.0586 (13)	0.0517 (11)	0.0326 (12)	0.0276 (10)	0.0273 (10)
C15	0.0711 (16)	0.0626 (14)	0.0490 (11)	0.0346 (13)	0.0290 (11)	0.0277 (11)
C16	0.0723 (18)	0.0768 (17)	0.0557 (13)	0.0328 (15)	0.0174 (12)	0.0290 (12)
C17	0.079 (2)	0.089 (2)	0.0744 (17)	0.0402 (18)	0.0249 (15)	0.0286 (16)
C18	0.107 (3)	0.155 (4)	0.082 (2)	0.077 (3)	0.041 (2)	0.059 (2)
C19	0.080 (2)	0.133 (3)	0.129 (3)	0.049 (2)	0.037 (2)	0.094 (3)
C20	0.077 (2)	0.105 (3)	0.113 (3)	0.036 (2)	0.0085 (19)	0.053 (2)
C21	0.071 (2)	0.104 (2)	0.0746 (18)	0.0330 (18)	0.0043 (15)	0.0319 (17)
C22A	0.110 (4)	0.102 (4)	0.141 (4)	0.048 (3)	0.050 (3)	0.074 (4)
C23A	0.134 (5)	0.165 (6)	0.138 (5)	0.078 (5)	0.078 (4)	0.103 (4)
C23B	0.134 (5)	0.165 (6)	0.138 (5)	0.078 (5)	0.078 (4)	0.103 (4)
C22B	0.110 (4)	0.102 (4)	0.141 (4)	0.048 (3)	0.050 (3)	0.074 (4)
F2	0.1159 (17)	0.0648 (11)	0.170 (2)	0.0284 (12)	0.0460 (15)	0.0509 (12)
O2	0.0988 (14)	0.0617 (10)	0.0562 (9)	0.0350 (10)	0.0394 (9)	0.0219 (8)
N4	0.0674 (13)	0.0624 (13)	0.0530 (10)	0.0330 (11)	0.0272 (9)	0.0285 (10)
N5	0.0665 (13)	0.0514 (12)	0.0492 (10)	0.0246 (11)	0.0224 (9)	0.0203 (9)
N6	0.0610 (12)	0.0545 (11)	0.0594 (11)	0.0257 (10)	0.0260 (9)	0.0255 (9)
C24	0.0588 (14)	0.0657 (15)	0.0608 (13)	0.0304 (13)	0.0192 (11)	0.0352 (12)
C25	0.0691 (17)	0.087 (2)	0.0766 (16)	0.0347 (16)	0.0277 (13)	0.0516 (15)
C26	0.0702 (19)	0.081 (2)	0.107 (2)	0.0257 (17)	0.0264 (16)	0.0609 (18)
C27	0.0718 (19)	0.0621 (17)	0.113 (2)	0.0248 (15)	0.0224 (17)	0.0439 (17)
C28	0.0696 (17)	0.0651 (16)	0.0833 (17)	0.0340 (15)	0.0228 (14)	0.0316 (14)
C29	0.0574 (14)	0.0590 (14)	0.0613 (13)	0.0304 (12)	0.0184 (11)	0.0300 (11)
C30	0.0543 (13)	0.0567 (13)	0.0512 (11)	0.0288 (11)	0.0167 (10)	0.0244 (10)
C31	0.0575 (14)	0.0513 (12)	0.0472 (11)	0.0281 (11)	0.0171 (10)	0.0176 (9)
C32	0.0635 (17)	0.091 (2)	0.0556 (13)	0.0333 (15)	0.0156 (12)	0.0278 (13)
C33	0.096 (2)	0.099 (2)	0.0509 (13)	0.0426 (19)	0.0214 (14)	0.0296 (14)
C34	0.099 (2)	0.0750 (18)	0.0646 (16)	0.0385 (17)	0.0440 (16)	0.0260 (14)
C35	0.0663 (18)	0.108 (2)	0.0825 (19)	0.0433 (18)	0.0366 (15)	0.0345 (17)
C36	0.0630 (16)	0.101 (2)	0.0584 (14)	0.0444 (16)	0.0183 (12)	0.0284 (14)
C37	0.0587 (14)	0.0600 (13)	0.0476 (11)	0.0311 (12)	0.0219 (10)	0.0284 (10)
C38	0.0591 (14)	0.0622 (14)	0.0477 (11)	0.0351 (12)	0.0238 (10)	0.0261 (10)
C39	0.0583 (14)	0.0600 (14)	0.0642 (13)	0.0306 (12)	0.0244 (11)	0.0315 (11)
C40	0.0717 (18)	0.0780 (18)	0.0615 (14)	0.0174 (15)	0.0172 (13)	0.0375 (14)
C41	0.090 (2)	0.0697 (17)	0.0641 (15)	0.0232 (16)	0.0301 (14)	0.0285 (13)
C42	0.0787 (19)	0.0749 (18)	0.0763 (17)	0.0306 (15)	0.0328 (14)	0.0414 (14)
C43	0.0750 (19)	0.0592 (15)	0.0782 (17)	0.0153 (14)	0.0270 (14)	0.0317 (13)
C44	0.089 (2)	0.0598 (16)	0.0830 (18)	0.0299 (15)	0.0360 (15)	0.0340 (14)
C45	0.095 (2)	0.088 (2)	0.091 (2)	0.0340 (19)	0.0387 (18)	0.0478 (18)
C46	0.127 (3)	0.143 (3)	0.094 (2)	0.058 (3)	0.057 (2)	0.069 (2)

### Geometric parameters (Å, º)

**Table:**

F1—C4	1.364 (4)	C21—H21B	0.9700
F2—C27	1.364 (5)	C21—H21A	0.9700
O1—C15	1.218 (3)	C22A—H22B	0.9700
O2—C38	1.220 (3)	C22A—H22A	0.9700
N1—C1	1.370 (3)	C22B—H22D	0.9700
N1—C14	1.378 (4)	C22B—H22C	0.9700
N2—C15	1.344 (4)	C23A—H23A	0.9600
N2—N3	1.384 (3)	C23A—H23C	0.9600
N3—C16	1.280 (5)	C23A—H23B	0.9600
N1—H1N	0.85 (3)	C23B—H23E	0.9600
N2—H2N	0.81 (3)	C23B—H23D	0.9700
N4—C37	1.378 (3)	C23B—H23F	0.9600
N4—C24	1.362 (4)	C24—C29	1.405 (3)
N5—C38	1.349 (3)	C24—C25	1.403 (5)
N5—N6	1.377 (4)	C25—C26	1.367 (5)
N6—C39	1.280 (3)	C26—C27	1.380 (5)
N4—H4N	0.85 (3)	C27—C28	1.368 (6)
N5—H5N	0.77 (2)	C28—C29	1.402 (5)
C1—C2	1.399 (5)	C29—C30	1.433 (4)
C1—C6	1.407 (3)	C30—C37	1.384 (3)
C2—C3	1.368 (5)	C30—C31	1.488 (3)
C3—C4	1.394 (5)	C31—C36	1.375 (5)
C4—C5	1.358 (5)	C31—C32	1.385 (4)
C5—C6	1.400 (3)	C32—C33	1.389 (4)
C6—C7	1.431 (4)	C33—C34	1.362 (5)
C7—C14	1.383 (3)	C34—C35	1.367 (4)
C7—C8	1.483 (3)	C35—C36	1.382 (5)
C8—C13	1.386 (4)	C37—C38	1.472 (4)
C8—C9	1.384 (4)	C39—C40	1.497 (4)
C9—C10	1.374 (5)	C39—C44	1.502 (4)
C10—C11	1.359 (5)	C40—C41	1.509 (5)
C11—C12	1.377 (6)	C41—C42	1.522 (6)
C12—C13	1.375 (5)	C42—C43	1.518 (4)
C14—C15	1.475 (3)	C42—C45	1.511 (5)
C16—C21	1.498 (5)	C43—C44	1.515 (5)
C16—C17	1.489 (4)	C45—C46	1.500 (5)
C17—C18	1.537 (6)	C25—H25	0.9300
C18—C19	1.491 (6)	C26—H26	0.9300
C19—C22A	1.545 (8)	C28—H28	0.9300
C19—C20	1.493 (6)	C32—H32	0.9300
C19—C22B	1.55 (2)	C33—H33	0.9300
C20—C21	1.503 (6)	C34—H34	0.9300
C22A—C23A	1.472 (10)	C35—H35	0.9300
C22B—C23B	1.48 (3)	C36—H36	0.9300
C2—H2	0.9300	C40—H40A	0.9700
C3—H3	0.9300	C40—H40B	0.9700
C5—H5	0.9300	C41—H41A	0.9700
C9—H9	0.9300	C41—H41B	0.9700
C10—H10	0.9300	C42—H42	0.9800
C11—H11	0.9300	C43—H43A	0.9700
C12—H12	0.9300	C43—H43B	0.9700
C13—H13	0.9300	C44—H44A	0.9700
C17—H17B	0.9700	C44—H44B	0.9700
C17—H17A	0.9700	C45—H45A	0.9700
C18—H18A	0.9700	C45—H45B	0.9700
C18—H18B	0.9700	C46—H46A	0.9600
C19—H19	0.9800	C46—H46B	0.9600
C20—H20B	0.9700	C46—H46C	0.9600
C20—H20A	0.9700		
			
C1—N1—C14	109.5 (2)	H22C—C22B—H22D	107.00
N3—N2—C15	120.6 (2)	C23B—C22B—H22C	108.00
N2—N3—C16	116.5 (2)	H23B—C23A—H23C	110.00
C14—N1—H1N	125.6 (18)	C22A—C23A—H23B	109.00
C1—N1—H1N	123.8 (18)	H23A—C23A—H23B	109.00
N3—N2—H2N	128 (2)	H23A—C23A—H23C	109.00
C15—N2—H2N	112 (2)	C22A—C23A—H23C	110.00
C24—N4—C37	109.2 (2)	C22A—C23A—H23A	109.00
N6—N5—C38	121.6 (2)	C22B—C23B—H23F	110.00
N5—N6—C39	116.6 (2)	C22B—C23B—H23E	110.00
C37—N4—H4N	120 (2)	H23D—C23B—H23F	109.00
C24—N4—H4N	129.1 (19)	H23E—C23B—H23F	110.00
C38—N5—H5N	110.6 (19)	H23D—C23B—H23E	109.00
N6—N5—H5N	127.1 (19)	C22B—C23B—H23D	109.00
N1—C1—C6	107.5 (2)	N4—C24—C25	130.3 (2)
N1—C1—C2	130.8 (2)	N4—C24—C29	108.2 (2)
C2—C1—C6	121.7 (2)	C25—C24—C29	121.6 (3)
C1—C2—C3	117.8 (3)	C24—C25—C26	117.5 (3)
C2—C3—C4	119.8 (3)	C25—C26—C27	120.5 (4)
F1—C4—C3	117.4 (3)	F2—C27—C28	118.8 (3)
C3—C4—C5	123.9 (3)	C26—C27—C28	123.9 (4)
F1—C4—C5	118.7 (3)	F2—C27—C26	117.3 (4)
C4—C5—C6	117.2 (2)	C27—C28—C29	116.7 (3)
C1—C6—C5	119.5 (2)	C28—C29—C30	133.1 (2)
C5—C6—C7	132.9 (2)	C24—C29—C28	119.8 (3)
C1—C6—C7	107.6 (2)	C24—C29—C30	107.1 (2)
C8—C7—C14	129.5 (2)	C31—C30—C37	127.6 (2)
C6—C7—C14	106.3 (2)	C29—C30—C37	106.3 (2)
C6—C7—C8	124.2 (2)	C29—C30—C31	126.1 (2)
C7—C8—C9	121.4 (2)	C30—C31—C36	123.0 (2)
C7—C8—C13	120.6 (3)	C32—C31—C36	117.6 (3)
C9—C8—C13	117.9 (3)	C30—C31—C32	119.4 (2)
C8—C9—C10	121.4 (3)	C31—C32—C33	121.0 (3)
C9—C10—C11	119.9 (4)	C32—C33—C34	120.1 (3)
C10—C11—C12	119.9 (3)	C33—C34—C35	119.7 (3)
C11—C12—C13	120.4 (4)	C34—C35—C36	120.3 (3)
C8—C13—C12	120.4 (4)	C31—C36—C35	121.3 (3)
N1—C14—C15	117.9 (2)	N4—C37—C30	109.3 (2)
N1—C14—C7	109.2 (2)	N4—C37—C38	117.9 (2)
C7—C14—C15	132.8 (2)	C30—C37—C38	132.8 (2)
O1—C15—C14	121.7 (2)	N5—C38—C37	115.6 (2)
N2—C15—C14	114.3 (2)	O2—C38—N5	122.5 (3)
O1—C15—N2	124.0 (2)	O2—C38—C37	122.0 (2)
N3—C16—C17	128.9 (3)	N6—C39—C44	116.5 (2)
C17—C16—C21	114.7 (3)	C40—C39—C44	115.4 (2)
N3—C16—C21	116.4 (3)	N6—C39—C40	128.1 (3)
C16—C17—C18	110.0 (3)	C39—C40—C41	113.3 (3)
C17—C18—C19	116.1 (3)	C40—C41—C42	113.4 (3)
C18—C19—C22A	117.0 (4)	C41—C42—C43	108.8 (3)
C18—C19—C22B	95.2 (8)	C41—C42—C45	113.4 (3)
C18—C19—C20	111.0 (4)	C43—C42—C45	113.1 (3)
C20—C19—C22A	108.7 (4)	C42—C43—C44	110.8 (3)
C20—C19—C22B	144.0 (10)	C39—C44—C43	112.3 (3)
C19—C20—C21	113.2 (4)	C42—C45—C46	114.0 (3)
C16—C21—C20	111.3 (3)	C24—C25—H25	121.00
C19—C22A—C23A	114.0 (6)	C26—C25—H25	121.00
C19—C22B—C23B	117.7 (17)	C25—C26—H26	120.00
C1—C2—H2	121.00	C27—C26—H26	120.00
C3—C2—H2	121.00	C27—C28—H28	122.00
C2—C3—H3	120.00	C29—C28—H28	122.00
C4—C3—H3	120.00	C31—C32—H32	119.00
C6—C5—H5	121.00	C33—C32—H32	119.00
C4—C5—H5	121.00	C32—C33—H33	120.00
C10—C9—H9	119.00	C34—C33—H33	120.00
C8—C9—H9	119.00	C33—C34—H34	120.00
C9—C10—H10	120.00	C35—C34—H34	120.00
C11—C10—H10	120.00	C34—C35—H35	120.00
C10—C11—H11	120.00	C36—C35—H35	120.00
C12—C11—H11	120.00	C31—C36—H36	119.00
C13—C12—H12	120.00	C35—C36—H36	119.00
C11—C12—H12	120.00	C39—C40—H40A	109.00
C12—C13—H13	120.00	C39—C40—H40B	109.00
C8—C13—H13	120.00	C41—C40—H40A	109.00
C18—C17—H17B	110.00	C41—C40—H40B	109.00
C16—C17—H17A	110.00	H40A—C40—H40B	108.00
C16—C17—H17B	110.00	C40—C41—H41A	109.00
C18—C17—H17A	110.00	C40—C41—H41B	109.00
H17A—C17—H17B	108.00	C42—C41—H41A	109.00
C17—C18—H18A	108.00	C42—C41—H41B	109.00
C17—C18—H18B	108.00	H41A—C41—H41B	108.00
C19—C18—H18A	108.00	C41—C42—H42	107.00
C19—C18—H18B	108.00	C43—C42—H42	107.00
H18A—C18—H18B	107.00	C45—C42—H42	107.00
C18—C19—H19	106.00	C42—C43—H43A	110.00
C22B—C19—H19	88.00	C42—C43—H43B	110.00
C20—C19—H19	107.00	C44—C43—H43A	109.00
C22A—C19—H19	107.00	C44—C43—H43B	109.00
H20A—C20—H20B	108.00	H43A—C43—H43B	108.00
C19—C20—H20A	109.00	C39—C44—H44A	109.00
C21—C20—H20B	109.00	C39—C44—H44B	109.00
C19—C20—H20B	109.00	C43—C44—H44A	109.00
C21—C20—H20A	109.00	C43—C44—H44B	109.00
H21A—C21—H21B	108.00	H44A—C44—H44B	108.00
C16—C21—H21B	109.00	C42—C45—H45A	109.00
C20—C21—H21A	109.00	C42—C45—H45B	109.00
C20—C21—H21B	109.00	C46—C45—H45A	109.00
C16—C21—H21A	109.00	C46—C45—H45B	109.00
C19—C22A—H22A	109.00	H45A—C45—H45B	108.00
C19—C22A—H22B	109.00	C45—C46—H46A	109.00
H22A—C22A—H22B	108.00	C45—C46—H46B	109.00
C23A—C22A—H22B	109.00	C45—C46—H46C	109.00
C23A—C22A—H22A	109.00	H46A—C46—H46B	109.00
C19—C22B—H22C	108.00	H46A—C46—H46C	110.00
C19—C22B—H22D	108.00	H46B—C46—H46C	110.00
C23B—C22B—H22D	108.00		
			
C1—N1—C14—C15	−176.4 (2)	N3—C16—C21—C20	−126.5 (4)
C14—N1—C1—C2	179.8 (3)	C16—C17—C18—C19	47.8 (5)
C14—N1—C1—C6	−0.9 (3)	C17—C18—C19—C22A	−175.1 (4)
C1—N1—C14—C7	1.3 (3)	C17—C18—C19—C20	−49.5 (6)
C15—N2—N3—C16	−175.1 (3)	C22A—C19—C20—C21	−178.0 (4)
N3—N2—C15—O1	2.9 (4)	C18—C19—C20—C21	51.9 (5)
N3—N2—C15—C14	−175.6 (2)	C18—C19—C22A—C23A	−65.3 (7)
N2—N3—C16—C21	−177.0 (3)	C20—C19—C22A—C23A	168.0 (5)
N2—N3—C16—C17	3.0 (5)	C19—C20—C21—C16	−54.1 (5)
C37—N4—C24—C29	−1.0 (3)	C25—C24—C29—C30	−178.4 (3)
C24—N4—C37—C38	178.8 (2)	N4—C24—C25—C26	179.1 (3)
C37—N4—C24—C25	178.4 (3)	C29—C24—C25—C26	−1.5 (5)
C24—N4—C37—C30	0.6 (3)	N4—C24—C29—C30	1.1 (3)
C38—N5—N6—C39	175.5 (3)	C25—C24—C29—C28	1.7 (4)
N6—N5—C38—O2	−6.1 (4)	N4—C24—C29—C28	−178.8 (3)
N6—N5—C38—C37	174.1 (2)	C24—C25—C26—C27	0.7 (5)
N5—N6—C39—C44	178.9 (2)	C25—C26—C27—F2	−178.8 (3)
N5—N6—C39—C40	−3.8 (4)	C25—C26—C27—C28	−0.1 (6)
N1—C1—C2—C3	−179.6 (3)	C26—C27—C28—C29	0.3 (6)
N1—C1—C6—C7	0.2 (3)	F2—C27—C28—C29	179.0 (3)
C6—C1—C2—C3	1.2 (5)	C27—C28—C29—C30	179.1 (3)
C2—C1—C6—C5	−1.1 (4)	C27—C28—C29—C24	−1.0 (4)
N1—C1—C6—C5	179.6 (2)	C28—C29—C30—C31	−3.0 (5)
C2—C1—C6—C7	179.6 (3)	C24—C29—C30—C37	−0.8 (3)
C1—C2—C3—C4	0.2 (5)	C28—C29—C30—C37	179.1 (3)
C2—C3—C4—F1	177.5 (3)	C24—C29—C30—C31	177.1 (2)
C2—C3—C4—C5	−1.9 (6)	C29—C30—C37—N4	0.1 (3)
C3—C4—C5—C6	2.0 (5)	C29—C30—C31—C32	−77.2 (4)
F1—C4—C5—C6	−177.4 (3)	C29—C30—C37—C38	−177.7 (3)
C4—C5—C6—C1	−0.5 (4)	C31—C30—C37—N4	−177.7 (2)
C4—C5—C6—C7	178.6 (3)	C37—C30—C31—C36	−76.8 (4)
C5—C6—C7—C14	−178.7 (3)	C37—C30—C31—C32	100.2 (3)
C5—C6—C7—C8	2.9 (5)	C31—C30—C37—C38	4.5 (4)
C1—C6—C7—C14	0.5 (3)	C29—C30—C31—C36	105.7 (3)
C1—C6—C7—C8	−177.9 (2)	C32—C31—C36—C35	−2.3 (5)
C8—C7—C14—N1	177.3 (3)	C30—C31—C36—C35	174.9 (3)
C6—C7—C8—C9	−78.4 (3)	C30—C31—C32—C33	−174.1 (3)
C6—C7—C14—N1	−1.1 (3)	C36—C31—C32—C33	3.1 (5)
C14—C7—C8—C13	−79.2 (4)	C31—C32—C33—C34	−1.8 (6)
C6—C7—C14—C15	176.2 (3)	C32—C33—C34—C35	−0.6 (6)
C6—C7—C8—C13	98.9 (3)	C33—C34—C35—C36	1.5 (6)
C14—C7—C8—C9	103.5 (4)	C34—C35—C36—C31	0.0 (6)
C8—C7—C14—C15	−5.5 (5)	N4—C37—C38—N5	164.4 (2)
C13—C8—C9—C10	−0.5 (4)	C30—C37—C38—O2	162.2 (3)
C9—C8—C13—C12	0.8 (4)	C30—C37—C38—N5	−17.9 (4)
C7—C8—C13—C12	−176.6 (3)	N4—C37—C38—O2	−15.5 (4)
C7—C8—C9—C10	176.8 (3)	N6—C39—C40—C41	139.8 (3)
C8—C9—C10—C11	−0.1 (5)	C44—C39—C40—C41	−42.9 (4)
C9—C10—C11—C12	0.4 (5)	N6—C39—C44—C43	−135.5 (3)
C10—C11—C12—C13	−0.1 (5)	C40—C39—C44—C43	46.9 (4)
C11—C12—C13—C8	−0.6 (5)	C39—C40—C41—C42	48.0 (4)
C7—C14—C15—O1	168.5 (3)	C40—C41—C42—C43	−56.4 (4)
C7—C14—C15—N2	−13.1 (4)	C40—C41—C42—C45	176.8 (3)
N1—C14—C15—N2	164.0 (2)	C41—C42—C43—C44	59.6 (4)
N1—C14—C15—O1	−14.5 (4)	C45—C42—C43—C44	−173.4 (3)
C21—C16—C17—C18	−49.2 (4)	C41—C42—C45—C46	−72.2 (4)
N3—C16—C17—C18	130.9 (4)	C43—C42—C45—C46	163.3 (4)
C17—C16—C21—C20	53.6 (5)	C42—C43—C44—C39	−55.4 (4)

### Hydrogen-bond geometry (Å, º)

Cg1, Cg3, Cg5 and Cg7 are the centroids of the N1/C1/C6/C7/C14, C8–C13, N4/C24/C29/C30/C37 and C31–C36 rings, respectively.

**Table:**

*D*—H···*A*	*D*—H	H···*A*	*D*···*A*	*D*—H···*A*
N1—H1*N*···O2	0.85 (3)	2.00 (3)	2.836 (3)	168 (3)
N4—H4*N*···O1	0.85 (3)	2.08 (3)	2.887 (3)	160 (2)
C17—H17*A*···N2	0.97	2.46	2.834 (5)	102
C40—H40*A*···N5	0.97	2.45	2.822 (4)	102
C9—H9···*Cg*1^i^	0.93	2.81	3.636 (3)	149
C17—H17*A*···*Cg*3	0.97	2.87	3.820 (3)	165
C40—H40*A*···*Cg*7	0.97	2.79	3.727 (4)	163
C40—H40*B*···*Cg*5^ii^	0.97	2.65	3.595 (4)	163

Symmetry codes: (i) −*x*+1, −*y*, −*z*+1; (ii) −*x*+1, −*y*+1, −*z*+2.
